# Structure of an ‘open’ clamp type II topoisomerase-DNA complex provides a mechanism for DNA capture and transport

**DOI:** 10.1093/nar/gkt749

**Published:** 2013-08-21

**Authors:** Ivan Laponogov, Dennis A. Veselkov, Isabelle M.-T. Crevel, Xiao-Su Pan, L. Mark Fisher, Mark R. Sanderson

**Affiliations:** ^1^Randall Division of Cell and Molecular Biophysics, 3rd floor New Hunt's House, Guy's Campus, King's College London, London, SE1 1UL, UK and ^2^Division of Biomedical Sciences, St. George's, University of London, Cranmer Terrace, London, SW17 0RE, UK

## Abstract

Type II topoisomerases regulate DNA supercoiling and chromosome segregation. They act as ATP-operated clamps that capture a DNA duplex and pass it through a transient DNA break in a second DNA segment via the sequential opening and closure of ATPase-, G-DNA- and C-gates. Here, we present the first ‘open clamp’ structures of a 3-gate topoisomerase II-DNA complex, the seminal complex engaged in DNA recognition and capture. A high-resolution structure was solved for a (full-length ParE-ParC55)_2_ dimer of *Streptococcus pneumoniae* topoisomerase IV bound to two DNA molecules: a closed DNA gate in a B-A-B form double-helical conformation and a second B-form duplex associated with closed C-gate helices at a novel site neighbouring the catalytically important β-pinwheel DNA-binding domain. The protein N gate is present in an ‘arms-wide-open’ state with the undimerized N-terminal ParE ATPase domains connected to TOPRIM domains via a flexible joint and folded back allowing ready access both for gate and transported DNA segments and cleavage-stabilizing antibacterial drugs. The structure shows the molecular conformations of all three gates at 3.7 Å, the highest resolution achieved for the full complex to date, and illuminates the mechanism of DNA capture and transport by a type II topoisomerase.

## INTRODUCTION

Type II DNA topoisomerases catalyse the transport of one DNA double helix through another in an ATP-dependent reaction (1–4). This manoeuvre allows the control of chromosomal DNA supercoiling and the removal of DNA supercoils, knots and catenanes generated in a variety of biological processes including DNA replication, transcription and recombination (1–4). Type II enzymes are ubiquitous in nature, are biologically essential and share structural and evolutionary features in common (5). Topoisomerase (topo) IV and gyrase are the type II enzymes expressed in bacteria (2,3). Topo IV mediates the unlinking of daughter chromosomes before cell division, whereas gyrase is unique in its ability to introduce negative supercoils into DNA thereby controlling chromosome supercoiling, which promotes replication fork advance and allows global regulation of gene expression. In each case, the active complex is a tetramer of two topo IV ParC and ParE subunits or gyrase GyrA and GyrB proteins. The ParC and GyrA subunits have an N-terminal DNA breakage-reunion domain linked to divergent C-terminal β-pinwheel domains that favour intermolecular DNA passage by topo IV causing DNA unlinking but intramolecular DNA transport by gyrase producing supercoiled DNA (6). By contrast, the N-terminal and C-terminal regions of the highly conserved ParE (GyrB) subunits form the ATPase- and Mg^2+^-binding-TOPRIM domains, respectively. These four functional domains are also present in eukaryotic topo II (2–4,7) but contained within each subunit of the homodimeric complex organized in a ‘GyrB-GyrA’ arrangement, i.e. N-terminal ATPase-TOPRIM-breakage/reunion-C-terminal domain. Thus, different type II topoisomerases share close functional and architectural similarities.

**Figure 1.**
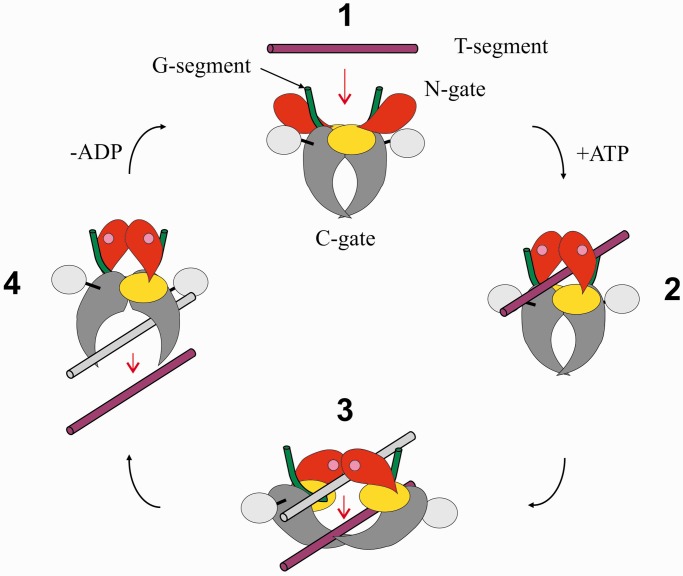
DNA strand passage by type II topoisomerases. A generic scheme is shown whereby a transported DNA segment (maroon) is passed through a transiently broken gate-DNA (green) by coordinated action of N- (or ATPase), DNA- and C-gates. The diagram shows the putative open (1) and closed clamp states (2–4) and putative conformational transitions driven by ATP binding/hydrolysis and ADP release (see text). ATPase and TOPRIM domains are shown in red and yellow, respectively. The DNA breakage-reunion and C-terminal β-pinwheel domains are depicted in grey and silver, respectively.

Early studies of *Escherichia coli* gyrase and eukaryotic topo II showed that the salient feature of type II topoisomerases is the formation of a transient DNA break involving a covalent-enzyme DNA intermediate termed the ‘cleavage complex’(8–10). Stabilization of the cleavage complex with antibacterial quinolones or anticancer topoisomerase inhibitors (11–14) has revealed that the DNA (known as the G-DNA or G-segment) contains a 4-bp staggered break formed by covalent attack and linkage of active site tyrosines, one to each 5′ phosphate end. DNA scission allows transport of a second DNA helix (known as the T-segment or transported DNA) through the break before resealing of the gate-DNA (2,3,8–10). It was argued that protein–protein contacts within the topoisomerase complex must exist to prevent inadvertent release of lethal DNA breaks and that the T-segment exits by passage between subunit interfaces (9,15). Evidence has accumulated that T-segment transport through the topoisomerase complex is coupled to the concerted opening and closure of two protein gates—the N-gate formed by the N-terminal ATPase domains and the C-gate formed within the ParC/GyrA region. These features have led to a generic mechanism for type II topoisomerases (Figure 1) (2,3).

It is proposed that the enzyme acts as an ATP-operated protein clamp that captures DNA for transport. First, the ‘open clamp’ state of the enzyme binds the gate-DNA in a highly bent conformation through interactions with the breakage-reunion and TOPRIM domains (**1** in Figure 1). Binding of ATP leads to dimerization of ATPase domains shutting the N-gate and capturing a T-segment in the ‘closed enzyme clamp’ (**2**). DNA capture allows transport through the opened DNA gate (**3**). Resealing of the DNA gate and opening of the C-gate facilitates release of the T segment (**4**). Finally, closure of the C-gate and release of ADP opens the N-gate converting the closed clamp (**4**) into the open clamp conformation (**1**) ready for another cycle.

The model describes the reaction mechanism but only in outline, and many key aspects of the reaction cycle are uncertain having been inferred by extrapolation from structures of individual domains trapped and stabilized using drugs, non-hydrolysable ATP analogues or suicide DNA substrates (16–19). In particular, the structures of the various open and closed clamp conformations of the enzyme and their sequential relationship in catalysis remain to be established. Available data from electron microscopy and small angle X-ray scattering are at very low resolution (20–22). Clearly, high-resolution X-ray crystal structures of full native topoisomerase II-DNA reaction intermediates will be essential in understanding the conformational transitions necessary for catalysis. A recent structure of yeast topoisomerase II (lacking the C-terminal domain) bound to a non-hydrolysable ATP analogue and suicide DNA substrate has provided information on a putative late-stage ‘closed’ enzyme clamp (23) (perhaps equivalent to state **3** in Figure 1). However, high-resolution structures for other reaction intermediates such as the open clamp state relevant to early events in catalysis have remained elusive.

To address this issue, we have focused on topo IV from *Streptococcus pneumoniae*, a Gram-positive human pathogen responsible for life-threatening infections, and for which topo IV is a target for clinically important anti-pneumococcal quinolones such as levofloxacin (24–26). Well-developed biochemistry for recombinant *S. pneumoniae* ParC and ParE subunits/domains (27–30) and strongly cleaved gate-DNA sequences such as the chromosomal E site (31) and plasmid V site (32) have made it an appealing system for mechanistic and X-ray crystallographic studies. Indeed, the first crystal structures for cleavage complexes of a type II topoisomerase were obtained with pneumococcal topo IV arrested on a 34 bp E-site DNA by quinolone or quinazolinedione antibacterials (18,19). Here, we describe X-ray crystal structures of the ‘open’ clamp conformation of *S. pneumoniae* topo IV, the first to be reported for any type II topoisomerase. One complex involving E-site DNA stabilized with levofloxacin was solved at 6.5 Å; a second structure containing V-site DNA and no drug was solved at 3.7 Å resolution, revealing two different DNA-binding sites. The latter complex shows the structure and disposition of all three gates at high resolution and has implications for topoisomerase mechanism and drug design. Based on these observations, we propose a plausible model for DNA capture and transport by type II topoisomerases.

## MATERIALS AND METHODS

### Proteins and DNA

Plasmid cloning, protein expression and purification of *S. pneumoniae* full-length ParE and ParC55 proteins have been described earlier (27,29). To produce the full-length ParE-ParC55 fusion protein, Vent DNA polymerase was used to amplify the *parE* gene using *S. pneumoniae* 7785 DNA as template. The forward primer (N7043) was 5′-AGGAGGTTCCATATGTCAAAAAAGG-3′ and the reverse primer (ParE NdeI-C) 5′-AAAACATATGAAACACTGTCGCTTCTTCTAGCGT-3′ each contained an NdeI restriction site (underlined). The PCR conditions were as follows: denaturation at 94°C for 1 min, annealing at 53°C for 1 min and polymerization at 72°C for 3 min. Reactions were performed over 30 cycles. The *parE* product was digested with NdeI and inserted into an NdeI-digested pET29a plasmid upstream of *parC* sequence encoding ParC55 that had been cloned as an NdeI-XhoI fragment. The resulting *parE-parC55* plasmid was transformed into *E. coli* BL21(λD3) pLysS, and the ParE-ParC55 fusion protein bearing a C-terminal His_6_ tag was overexpressed and purified to >95% homogeneity by Ni-chelate chromatography. The fusion protein was catalytically active promoting gemifloxacin-induced DNA cleavage (see Supplementary Figure S5) and exhibited the expected basal level of DNA relaxation activity (Crevel, I., Pan,X.S. and Fisher,L.M., results not shown).

DNA oligomers were synthesised by solid-phase phosphoramidite chemistry and doubly high performance liquid chromatography purified by Metabion, Munich.

### Crystallization and data collection

#### Low-resolution complex (space group H32)

**Figure 2.**
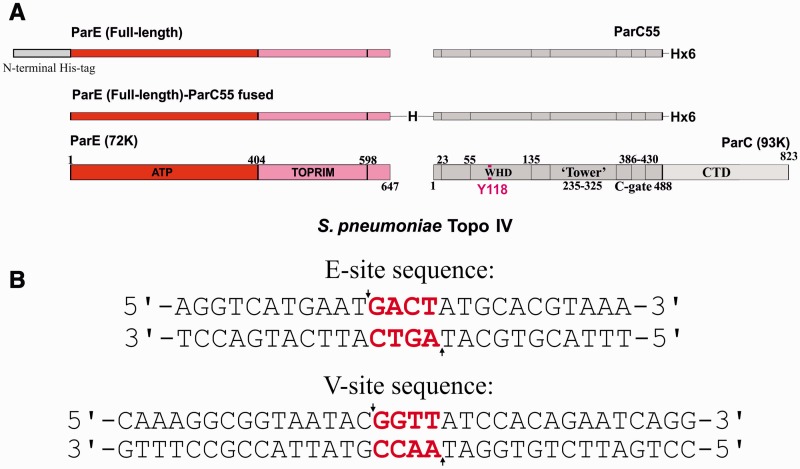
*Streptococcus pneumoniae* topo IV and its E and V cleavage sites on DNA. (**A**) Domain structures of 72 K ParE and 93K ParC subunits of *S. pneumoniae* topo IV. Figure shows the recombinant non-fused (top), fused (middle) and native (bottom) ParE and ParC subunits. ATP and TOPRIM denote N-terminal ATPase and C-terminal Mg^2+^ binding domains of ParE. CTD denotes the DNA binding C-terminal domain of ParC that orients DNA for transport and which adopts a β-pinwheel structure. WHD indicates the winged helix domain. Numbers denote amino acid residues in the 647 residue ParE and 823-residue ParC proteins. (**B**) DNA sequences of the 34mer V-site and 26mer E-site used in crystallization studies. The 4-bp overhanging sequence which is generated upon cleavage between −1 and +1 nucleotides (arrows) is shown in red.

The low-resolution complex crystals of ParC55/ParE full-length (N-terminally tagged)/26mer E-site DNA/levofloxacin were grown by the sitting drop technique in standard MRC 96-well plates. First, the proteins were dialysed overnight against 20 mM Tris–HCl (pH 7.5), 1 mM β-mercaptoethanol, 0.05% sodium azide and 200 mM sodium chloride, then their concentrations were measured and the 1:1 molar ratio mixture was formed (see Figure 2A for domain composition of the proteins used). After that the protein mixture was concentrated to 4 mg/ml, and the dialysis was repeated overnight against buffer of the same composition but with a salt concentration of 100 mM sodium chloride in the second step. Then, 10 mM MgCl_2_, 2 mM levofloxacin and 1:1.2 molar ratio of 26-mer E-site DNA (see Figure 1B for E-site sequence) were added (final concentration). The mixture was pre-incubated overnight at room temperature. The crystallization drops were formed by mixing 600 nl of the protein mixture with 400 nl of reservoir solution [50 mM sodium cacodylate (pH 6.5–7.0), 2.5% Tacsimate™ (pH 7.0) (Hampton Research Corp.) (33), 100 mM sodium chloride, 7.5 mM magnesium chloride, 3–4% isopropanol]. Crystals formed after several weeks at room temperature. The best crystals were flash-cooled to 100 K into a cryoprotectant buffer [50 mM Na cacodylate (pH 6.5), 2.5% Tacsimate™ (pH 7.0) (Hampton Research Corp.) (33), 62.5 mM KCl, 7.5 mM MgCl_2_, 1 mM β-mercaptoethanol, 30% (v/v) MPD]. Crystals were formed in space group H32 with cell dimensions a = b = 213.58 Å, c = 211.83 Å, α = β = 90° and γ = 120°, the best overall resolution was 6.53 Å (Supplementary Figure S1). Data collection was performed at the Diamond synchrotron (Oxfordshire, UK) using the 3rd generation beamline I03, equipped at that time (2010) with an ADSC detector. The wavelength for the data collection was set to 0.9763 Å. Data were integrated and reduced using Xia2 (XDS) (34–36).

#### High-resolution complex (space group P4_2_2_1_2,)

Full-length ParE-ParC55 fusion protein (see Figure 2A for domain composition of the protein used) at 3 mg/ml concentration was dialysed against 20 mM Tris–HCl (pH 7.5), 1 mM β-mercaptoethanol, 0.05% sodium azide and 200 mM or 100 mM sodium chloride (initial and final steps, respectively). The dialysed protein was mixed with the 34 bp V-site DNA (see Figure 2B for V-site sequence) at 1:2.2 protein: DNA molar ratio. After that, 10 mM magnesium chloride and 2 mM levofloxacin (final concentration) were added, and the crystallization mixture was incubated overnight at room temperature. Crystallization was performed by conventional sitting drop vapour diffusion in 96-well MRC crystallization plates (drop ratio: 600 nl + 400 nl protein + reservoir, respectively). Crystals grew after 7 days from 50 mM sodium citrate (pH 5.0), 4–7% isopropanol and were also hard to reproduce due to strong sensitivity to the pH of the crystallization solution—our subsequent experiments showed that deviation of ± 0.1–0.15 pH units was sufficient to step-over the crystallization conditions. The crystals were flash frozen into a solution of 50 mM sodium citrate (pH 5.0), 60 mM sodium chloride, 30% MPD, 4% isopropanol, 1 mM β-mercaptoethanol. Crystals were formed in space group P4_2_2_1_2 with cell dimensions a = b = 160.384 Å, c = 280.806 Å, α = β = γ = 90°, the best overall resolution was 3.7 Å (Supplementary Figure S2). High-resolution data collection was performed at the Diamond synchrotron (Oxfordshire, UK) using 3rd generation beamlines I04-1 and I24 equipped with Pilatus detectors (2012). The wavelength for data collection was set to 0.92000 Å. Data were integrated and reduced using Xia2 (XDS) (34–36).

### Structure determination

Structures were solved by molecular replacement in Phaser (35,37) using as search models our ParC55/ParE30/18mer DNA+levofloxacin structure (PDB ID: 3RAE) as a starting model and a ParE ATP domain homology modelled in 3D-JigSaw (38) on the basis of the structure of the ATPase domain of the *E. coli* gyrase (PDB ID: 1EI1) (39).

The 34 bp DNA model from the structure of the *Saccharomyces cerevisiae* (PDB ID: 2RGR) (17) was found to be useful for extending our 18mer DNA model for the high-resolution structure. In the high-resolution structure, the second DNA molecule was discovered during the initial refinement and has been positioned into difference Fourier density maps. As a consequence of the V-site not having a palindromic sequence, and the fact that the second DNA lies on a crystallographic 2-fold symmetry axis, it was modelled using two alternative orientations with fixed occupancies of 0.5 (Supplementary Figure S3).

The low-resolution structure was initially refined in CNS-Solve (40) using the DEN refinement protocol (41). Subsequent refinement was performed in Phenix (42). However, despite heavy restraints, the structure looked over-fitted with gaps between R and R-free exceeding 10%, which is not surprising at such low resolution and without a good starting model for the ATPase domain of ParE. Therefore, for the final deposited structure in space group H32, the refined protein model from our high-resolution structure in space group P4_2_2_1_2 was used to retrofit the data and refined with rigid body and TLS methods in Phenix (42) (no individual xyz refinement performed). This approach allowed for the final R/R-free of 0.2474/0.2982 to be obtained, whereas no significant changes to the electron density maps were detected. The model was inspected and manually corrected using the graphics program WinCoot (43). The bound levofloxacin drug molecules could easily be seen in the F_obs_-F_calc_ map and thus were added to the final model. According to ProCheck (44), 774 residues (86.4%) are in most favoured regions of Ramachandran plot, 108 residues (12.1%) are in additional allowed regions, 9 residues (1.0%) are in generously allowed regions and 5 residues (0.6%) are in disallowed regions.

As for the high-resolution structure in space group P4_2_2_1_2, refinement was performed in Refmac (35,45,46) during the initial stages using jelly body refinement, gradually increasing the resolution starting from 4.5 Å in steps of 0.5 Å. Subsequent refinement was executed in Phenix (42) using the secondary structure restraints derived from the high-resolution structure of ParC55/ParE30/18mer+levofloxacin (PDB ID: 3RAE). Multiple rounds of rigid body, positional and TLS refinement were performed, and the model each time was inspected and manually corrected using the graphics program WinCoot (43) after the refinement cycle. According to ProCheck (44), 1511 residues (82.7%) are in most favoured regions of Ramachandran plot, 277 residues (15.2%) are in additional allowed regions, 24 residues (1.3%) are in generously allowed regions and 16 residues (0.9%) are in disallowed regions.

The final data collection refinement statistics are given in Supplementary Table S1.

In both low- and high-resolution structures, it was not possible to trace clearly the residues 400–413 of ParE, which form a flexible hinge joining ATPase and TOPRIM domains owing to their high mobility. However, the absence of extra electron density observed above the bound G-segment DNA, the significantly longer distance from residue 399 to residue 414 of ParE on the opposite side of the G-segment DNA-binding groove (30.5 Å) compared with the distance to the identical residue 414 on the same side (15.5 Å), and the requirement that the G-gate be able to approach and bind in the groove allowed us to conclude that the ATPase domains are linked in *cis* to the TOPRIM domains to which they are closest (i.e. lie on the same sides of the DNA binding groove of ParC55). This is in contrast to the criss-cross (*trans*) conformation observed in the ADPNP-closed structure reported by Schmidt *et al.* (23).

### Manuscript preparation

**Figure 3.**
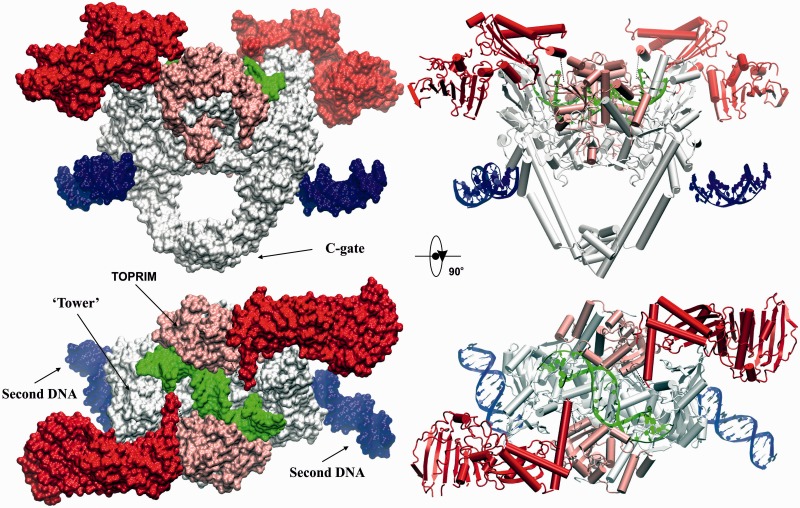
High-resolution ‘open clamp’ structure of *S. pneumoniae* topoisomerase IV bound to 34mer V-site DNA. The surface (left) and cartoon (right) representations of the dimeric complex of fused full-length ParE-ParC55 are shown in orthogonal views. ParC55 is in silver, ParE N-terminal gate domain (including the ATPase subdomain) is in red, ParE TOPRIM domain is in light pink, G-segment DNA is in green, the second DNA bound to α-helices near the C-gate is in blue.

Initial model manipulations in preparation for movie generation were performed in WinCoot (43). Movies were generated using custom-developed (by I.Laponogov) script for CNS-Solve (40). The 3D representations of the molecules were generated using VMD/Internal Tachyon renderer (47) (Figures 3, 7–10 and Supplementary Movies S1 and S2) or PyMOL (The PyMOL Molecular Graphics System. Schrödinger, LLC.) (Figures 5, 6 and 8, Supplementary Figures S1–S4, Supplementary Movie S1). The core r.m.s.d. values were calculated using WinCoot (SSM) (43), crystallographic unit cell formation was visualized in MapView (crystal lattice visualization software developed by I.L. in our group) and rendered using Pov-Ray (http://www.povray.org/). Movie assembly was performed using SSMM (Slide Show Movie Maker published by Joern Thiemann as freeware) with subsequent editing in Corel VideoStudio Pro X3©. Figures were assembled and prepared using CorelDraw X5©.

**Figure 4.**
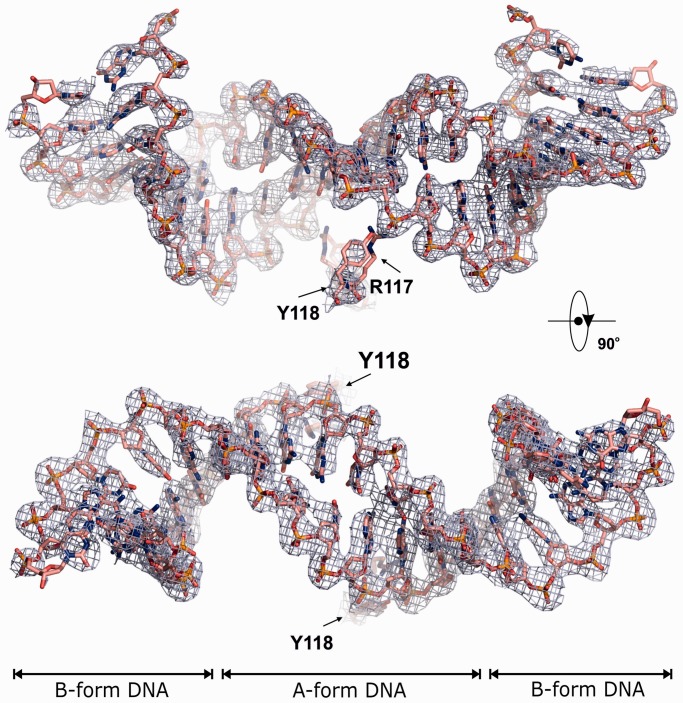
G-segment DNA in the high-resolution structure. Orthogonal views of the bound G-segment V-site DNA with the 2F_obs_-F_calc_ electron density map at 1.5σ overlapped and the corresponding active site tyrosines-118 and arginines-117 displayed. The DNA form is indicated according to w3DNA (48).

**Figure 5.**
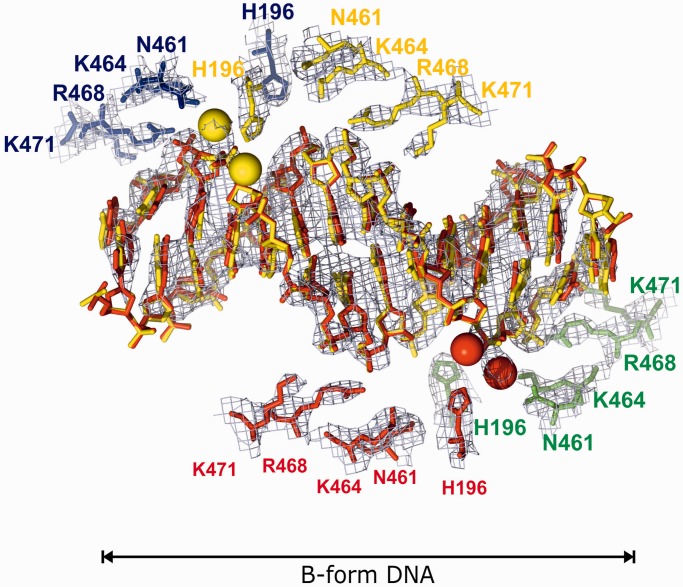
ParC subunit interactions with the second DNA duplex. Four topo IV dimers coordinate the B-form DNA duplex in the crystal with the corresponding ParC residues involved are shown in red, yellow, green and blue. Green and red spheres show the positions of water molecules. The DNA form is indicated according to w3DNA (48).

**Figure 6.**
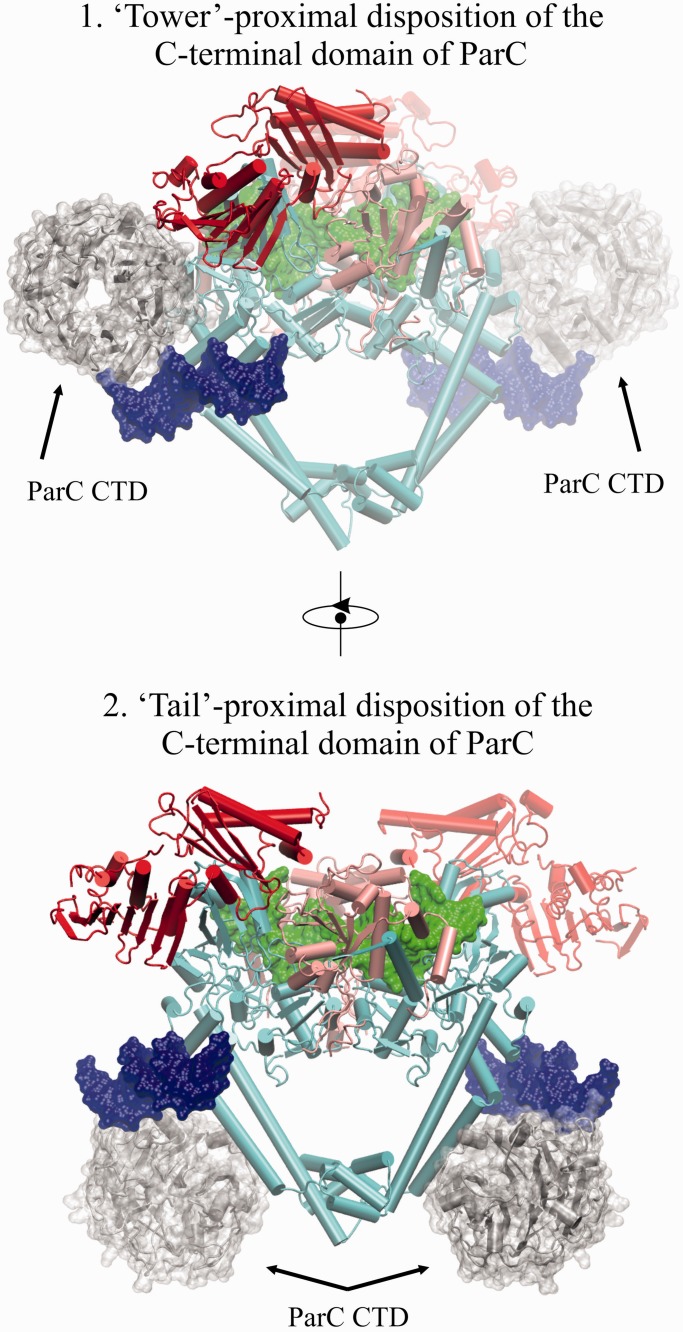
Putative binding positions of the modeled C-terminal β-pinwheel domain of ParC relative to the solved biological dimer and the second DNA-binding site. ParC55 is in cyan, ParE N-terminal gate domain (including the ATPase subdomain) is in red, ParE TOPRIM domain is in light pink, G-segment DNA is in green, the second DNA bound to α-helices near the C-gate is in blue. The model of the C-terminal β-pinwheel domain of ParC is in silver with additional semi-transparent surface representation.

**Figure 7.**
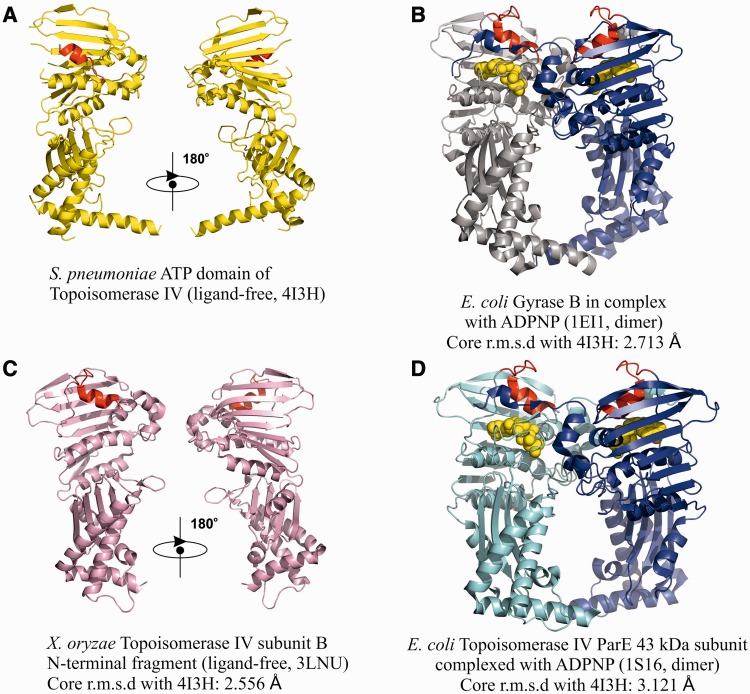
The ATPase domains. Shown are cartoon representations of the ATPase domains of ParE or GyrB in *S. pneumoniae* (top left), *E. coli* [right top and bottom for GyrB (39) and ParE (49), respectively) and *X. oryzae* ParE (Heo, Y.-S. Crystal structure of ParE subunit. PDB code: 3LNU). Where applicable, the domains are in their dimerized form stabilized by ADPNP, a non-hydrolysable ATP analogue (displayed in Licorice mode). An ‘ATPase-active site covering’ α-helix present in *S. pneumoniae* ParE and its analogue in other structures is shown in red. ADPNP in B and D is shown in gold.

**Figure 8.**
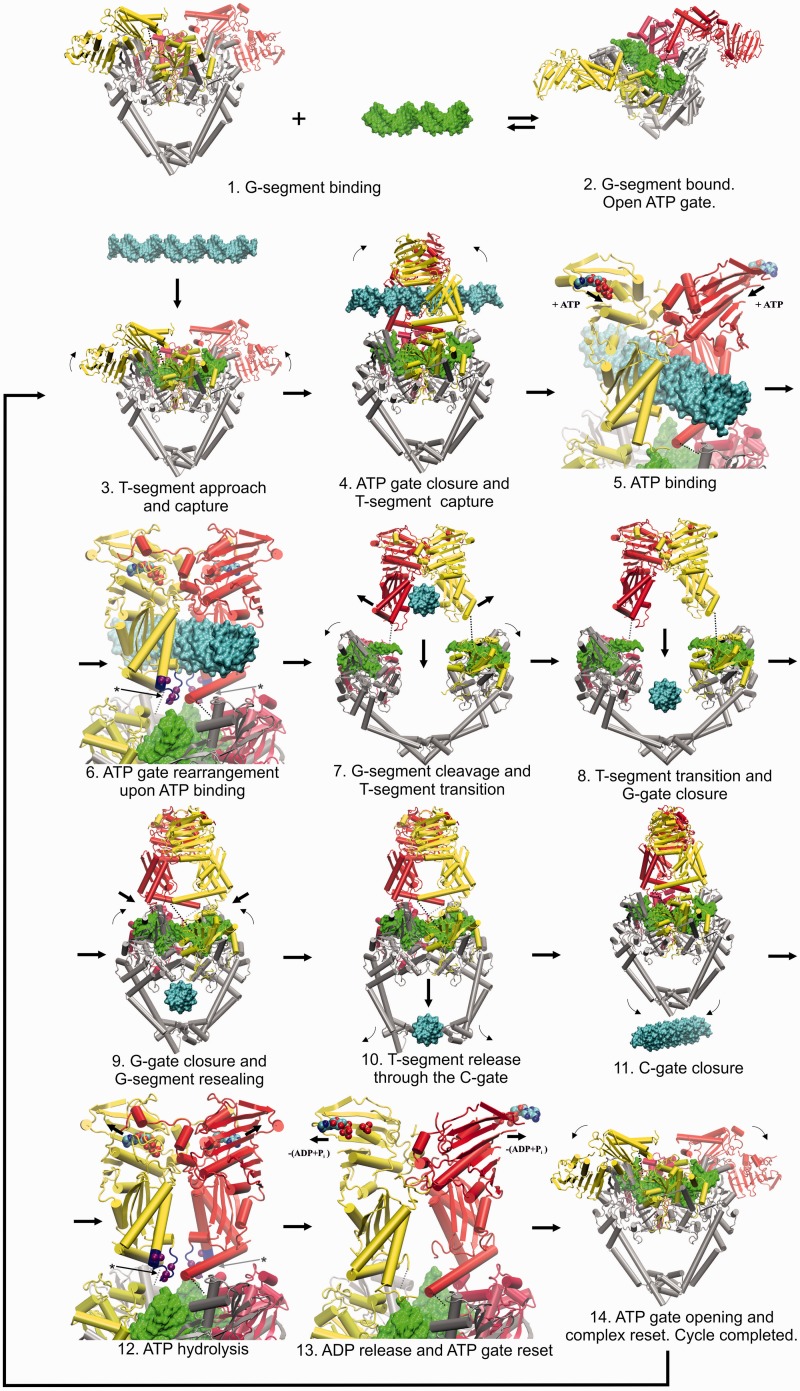
Proposed model for the T-segment DNA capture and transport based on the high-resolution structure of the open N-gate topoisomerase complex with the V34 G-segment DNA. The individual distinct stages are numbered 1 to 14. The protein is shown in Cartoon representation, the DNA is shown in surface representation. The ParE ATPase and TOPRIM domains are shown in red/light red for one side and yellow/light yellow for the other one, with dotted lines indicating the flexible poorly ordered protein pivot corresponding to *S. pneumoniae* ParE residues 399–413. The G-segment is in green, the T-segment is in cyan and ParC55 is in silver. ATP/ADP + leaving phosphates are shown in van der Waals representation and coloured according to the atom type. Residues 303–316 of ParE comprising the K-loop (23) are shown in blue where applicable and the corresponding arginine/lysine Cα-s are shown in purple using van der Waals representation (stages 6 and 12, indicated by asterisks and arrows). Small arrows indicate general movements of the domains, whereas the larger arrows indicate progress through the topo II cycle. The open clamp state (stage 2 and 14) and its conversion to the closed clamp form (stage 5) are new findings from our study. There is uncertainty about exactly how the two ATP molecules are used and whether all type II topoisomerase systems follow the same hydrolytic mechanism. For simplicity and to focus on the pathway of DNA transport, we show both ATP molecules hydrolyzed at stage 12, though one molecule could be hydrolysed earlier (at stage 7 and 8) as postulated for yeast topo II (2,3).

**Figure 9.**
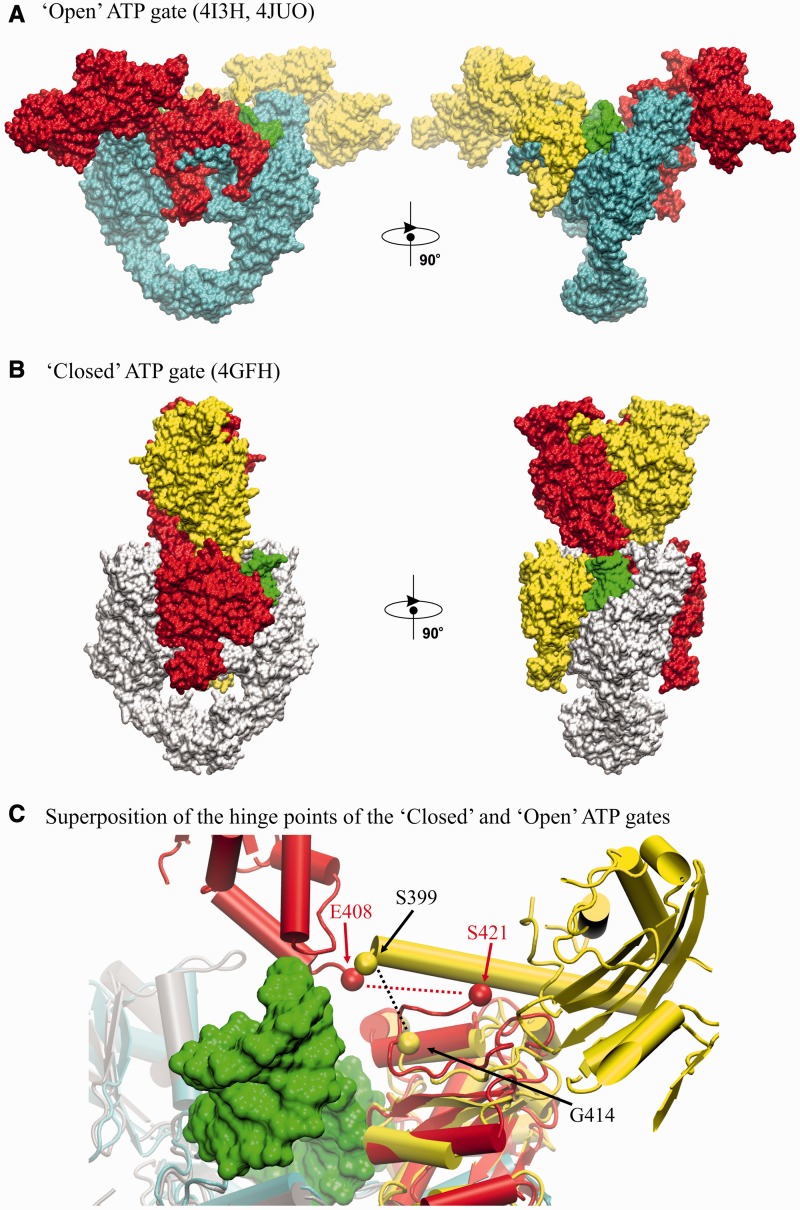
‘Open’ and ‘closed’ ATP gate conformations known from crystallographic structures. (**A**) ‘open’ ATP gate structure of pneumococcal topo IV (this work); (**B**) ‘closed’ ATP gate structure of yeast topo II (23). The structures are displayed in ‘surface’ mode in orthogonal views. Corresponding ParE domains are shown in red/yellow. G-segment is in green. ParC55 domains in (A) are in cyan. ParC55-equivalent domains in (B) are in white. (**C**) Diagram superimposing the hinge regions of open and closed clamps of type II topoisomerases. For the open clamp topo IV structure, linkage of ATPase and TOPRIM domains (shown in yellow) through their respective ParE S399 and G414 residues (black dotted line) occurs on the same side of the complex, whereas for the closed clamp of yeast topo II, the flexible hinge linking E408 and to S421 (red dotted line) passes over the bound DNA (shown in green) to connect ATPase and TOPRIM domains on opposite sides of the gate.

**Figure 10.**
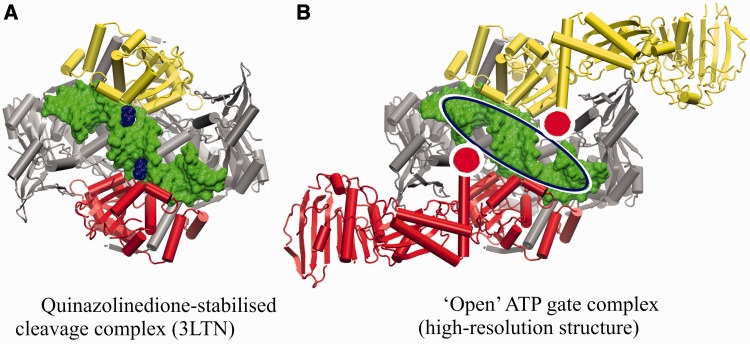
Structural implications for rational drug design. (**A**) Previously published cleavage complex of topo IV from *S. pneumoniae* stabilized by PD 0305970 (19) with the bound drug molecules shown in blue. (**B**) Corresponding view of the high-resolution structure of topo IV from *S. pneumoniae* comprising the full-length ParE domains. ParC55 is in silver, G-segment DNA is in green, ParE is in red/yellow. The area available for rational drug design exploration and exploitation is indicated by the blue/white ellipse. The locations of the flexible pivot links between the ATPase and TOPRIM domains of ParE are in white/red circles.

### Data deposition

Atomic coordinates and structure factors have been deposited in the PDB with accession codes 4I3H (high-resolution structure, space group P4_2_2_1_2) and 4JUO (low-resolution structure, space group H32).

## RESULTS AND DISCUSSION

### ‘Open' ATP gate structure of topo IV from *S.pneumoniae*

Our initial attempts to crystallize a three-gate open clamp complex of *S. pneumoniae* topo IV used recombinant full-length ParE protein and a ParC55 protein comprising the breakage-reunion domain of ParC (Figure 2A). Crystallization trials were set up with differently sized oligonucleotide duplexes in the presence and absence of quinolones. A levofloxacin-DNA cleavage complex of ParE and ParC55 proteins with a 26mer E-site DNA (Figure 2B) yielded a crystal structure in the rhombohedral space group H32 with a best resolution of 6.5 Å (Supplementary Figure S1, Supplementary Table S1). Though at low resolution, the structure clearly showed an open clamp conformation with undimerized ParE ATPase domains and clear conformations and dispositions of the ATPase (N)-gate, the drug-bound DNA-gate and C-gates (Supplementary Figure S1, PDB entry 4JUO). To obtain a structure at higher resolution, we explored a number of modifications to the crystallization protocol. First, to prevent subunit dissociation and generate a stabilized complex, we engineered a catalytically active fusion protein comprising the full-length ParE and breakage-reunion ParC55 domain (Figure 2A and Supplementary Figure S5). Second, we used an alternative gate-DNA, namely, a 34mer DNA duplex comprising the plasmid V-site (Figure 2B), which we have shown is also strongly cleaved by pneumococcal topo IV (Arnoldi ref). Third, we experimented with 1:1.2 and 1:2.2 protein–DNA ratios. Co-crystallization of the fusion protein with a 2.2-fold excess of V-site DNA yielded a crystal structure in space group P4_2_2_1_2 (Supplementary Figure S2) with the best resolution of 3.7 Å. This new structure reveals a novel (ParE-ParC55)_2_ dimer complex bound to two DNA molecules and adopting the open clamp conformation (open ParE N-gate; closed G and C gates) (Figure 3, Supplementary Movie S1). Essentially, the same open clamp conformation and domain dispositions were seen for this drug-free complex as was observed for the levofloxacin-stabilized 6.5 Å complex obtained in a different space group using a different gate-DNA sequence and different crystallization conditions (Supplementary Figures S1–S4).

From the high-resolution structure, the complex resembles a bull’s head with the open N-gate forming the horns (Figure 3, Supplementary Figures S2 and S4, Supplementary Movie S1). The overall dimensions of the complex are ∼190 Å× 90 Å× 120 Å and the total molecular weight exceeds 287 kDa (counting 1/2 of the second DNA molecule in Figure 3). The DNA bound at the G-gate is bent and uncleaved and adopts an A-form conformation immediately at the scission sequence while retaining B-form on the flanks (Figure 4), as seen previously for drug-free DNA complexes of ParC55 with ParE30 (19). By contrast, the second DNA molecule links each of four complexes together within the crystal lattice by binding to the long helices of the C-gate via ParC R468, K464, N461 and H196 residues (Figure 5, Supplementary Figure S3) and is entirely in the linear unbent B-form (Figure 5). Evidently, two different conformational states of the V-site gate-DNA co-exist within the crystal under the same conditions. Thus, in answer to a longstanding question, it appears that the DNA gate sequence is linear but undergoes both enzyme-induced bending and helical transition from linear B- to a bent B-A-B-form on binding as a DNA gate. The results support the suggestion that distortion and bending of the DNA is required for DNA scission at the gate (19).

### Probing the non-gate DNA-binding site

The presence of a second DNA-binding site on ParC has not been reported previously, and its unveiling is most likely a consequence of the higher ratio of DNA used in the crystallization conditions. The function of the site is unknown but through DNA connectivity, it could in principle have a role in G-gate stabilization or T-segment alignment. In particular, the site is of interest in relation to the position and orientation of the C-terminal β-pinwheel domain of topo IV, which is believed to facilitate the T-segment orientation and transition through the reversibly cleaved G-segment. In the case of gyrase, DNA adjacent to the G-segment DNA is bent around the β-pinwheel aligning it as a T-segment, ensuring intramolecular DNA transport and resulting in negative supercoiling of the DNA (1–4,9). However, the disposition of the β−pinwheel domain has yet to be established precisely in the context of a full-length type II topoisomerase-DNA complex. Our preliminary modelling showed that the location of the second bound DNA is favourable for the DNA interaction with the modelled C-terminal domain of topoisomerase II in both of its plausible locations (Figure 6) according to the accepted paradigm (1–4) as well as to the results of the low-resolution SAXS studies of gyrases (21,22). Of the four ParC residues engaged in non-gate DNA-binding lie on the enzyme surface, and that K464 and K471 (but not N461 and R468) are conserved among ParC and GyrA proteins of *E. coli, Staphylococcus aureus and S. pneumoniae*, suggesting functional significance (data not shown). To test this aspect, we made a number of full-length ParC proteins in which one or more residues in the second DNA binding were altered to alanine or negatively charged glutamate and tested their catalytic activity. Mutant *S. pneumoniae* ParC (K464A), ParC (R468A) and triple mutants ParC (K464A,R468A,K471A) and ParC (K464A, R468E, K471E), all exhibited wild-type activities in DNA decatenation, DNA relaxation and DNA cleavage assays when reconstituted with ParE (Pan, X-S., Sarigul, M. and Fisher, L.M., results not shown). Thus, although the second DNA coordinates four topo IV dimers in the crystal, mutagenesis of this interface did not appear to alter catalytic activity, with the proviso that we did not examine relaxation of positively supercoiled DNA, another topological form discriminated by topo IV. Moreover, we cannot presently rule out a biological role involving higher-order structure through concerted coordination of different topo IV molecules.

### ATPase domains

The complex was crystallized in the absence of ATP and reveals novel aspects of the open N-gate conformation (Figure 3). The two N-terminal ParE ATPase domains (residues 1–399 shown in red in Figure 3) (that dimerize on ATP binding to close the N-gate) are located far apart fully exposing the G-segment (in green) to the solvent from above (Figure 3, Supplementary Movie S1). The ATPase subdomains (residues 1–220) are located distal to the main body of the complex, with the rest of the gate (residues 221–399) sitting on top of the core complex with long α-helices (ParE residues 373–399) occupying the groove between the ParC55 ‘Tower’ (shown in silver) and the ParE TOPRIM domain (shown in light pink) with their polar side chains mainly exposed to solvent above the complex suggesting a possible role in T-segment capture. The overall domain orientation resembles that predicted for *E. coli* gyrase in solution using low-resolution SAXS data (21,22) and electron microscopy (20). The N-terminal ParE domains were easily traceable with the exception of a few disordered loops (Figure 7A and C for the analogous *Xanthomonas oryzae* ATPase domain), which are ordered in previously solved ADPNP-induced dimers of ATPase domains examined in isolation (39,49) (Figure 7B and D, Supplementary Movie S2). These regions include the first 19 amino acids of ParE, which participate in interdomain linkage on ATP binding and help form the nucleotide binding site. In our structure, the ATP binding site is empty and covered by the neighbouring α-helix from the same ParE monomer, plausibly protecting it from ATP binding before T-segment capture (Figure 7A), a feature not previously observed in other crystal structures of the ATPase domains of ParE and GyrB dimers (Figure 7B–D) (39,49).

Interestingly, the N-terminal gate is connected to the TOPRIM domain (residues 400–647) of ParE via a single flexible linkage comprising residues 400–413 bridging a distance of 15.5 Å (Figure 3). The protein link allows for pivoting and dimerization of ParE domains necessary for N-gate closure. The link is mobile and poorly ordered, so its exact fold is not evident from the crystallographic data. However, it is clear that in the open clamp conformation, linkage of the N-gate domains to the TOPRIM domains of ParE occurs on the ‘same’ sides of the heterodimer (see Figure 3 and ‘Materials and Methods’ section for explanation) and does not criss-cross between the two sides of the complex, which would otherwise impede DNA transport (Figure 1).

### A mechanism for DNA capture and transport

The new features revealed in the open enzyme conformation together with available partial structures suggest a plausible model for DNA capture and strand passage by the complex (shown in Figure 8 and animated in Supplementary Movie S2). Initially, the ‘open and welcoming’ conformation of the ATPase domains (Figure 3) allows for the unhindered approach of the G-segment DNA, which on binding undergoes distortion from linear B-form to the bent and extended A-form DNA gate (Figures 3, 4 and 8, stages 1–3). Next, the T-segment approaches (perhaps pre-coordinated by the ‘Towers’ and the long α-helices of the ATPase domains) and undergoes transient capture by upward ∼90° rotation of t he ATPase domains around the pivoting points, which shifts them closer by a few Ångstroms [in accordance with conformation of the ADPNP-stabilized GyrB NTD dimer from *E. coli* gyrase, PDB code: 1EI1, which was used to model the closed ATPase domains with ATP bound (39)] (stage 4). On capture, the T-segment will be located in the middle cavity of the ATPase domain dimer between the ATPase site at the top and the crossed long α-helices (residues 373–399) at the bottom (Figure 8, stage 4–6). This cavity is large enough to accommodate a captured DNA duplex and has the α-helices comprising residues 285–305 and 317–321 at the exact distance and position to intercalate into the major groove of the DNA, thus stabilizing binding (stage 5). Sensing the presence of both the T-segment and G-segment (relayed by the positively charged K-loop (23), a conformational transducing element in the ATPase domain critical for catalysis), the ATPase domains recruit ATP and undergo rearrangement of several loops in the active site, particularly the known cross interpolation of the first 20 residues of ParE, forming the ATP-binding site and locking the dimerized ATPase domains together (stage 6) as shown in Figure 8 and Supplementary Movie S2 (However, at this point, it is not clear if the ATP needs to be pre-bound to the incomplete binding site to ‘prime’ the ATP domains and make them ready to capture the T-segment or the capture happens first and then the ATP binds and locks the assembly). These events tighten the whole arrangement prompting DNA cleavage and opening of the G-gate formed by the ParC55 and ParE TOPRIM domains (stage 7) (modelled on the opened conformation of full-length ParC from *E. coli*, PDB code: 1ZVU) (6). DNA cleavage is aided by the strain and torsional stress present in the helically distorted and bent gate-DNA facilitating attack by active-site tyrosines (Y118 of ParC). When the DNA-gate opens sufficiently to pass the T-segment (∼36 Å in our model), the simultaneous spring action of the long α-helices of the ATPase domains rotate the T-segment by ∼45^°^, presenting it almost perpendicular to the G-segment (stage 7). This domain rearrangement also moves the long ParE α-helices previously blocking the T-segment out of the way allowing DNA transport through the DNA gate. Once the T-segment reaches the lower cavity in the heterotetramer next to the C-gate (stage 8), the G-gate closes and is resealed (stage 9) coordinated with T-segment release via the opening and closure of the C-gate (stages 10 and 11). Closure of the G-gate is achieved most simply by reverse 45° rotation of the ATPase domain dimer bringing it back to the original position with the K-loop contacting the G-segment, inducing ATP hydrolysis, releasing ADP and resetting the ATPase domains back to the open conformation (stages 12–14) (Figure 8, Supplementary Movie S2).

### Interconversion between ‘open’ and ‘closed’ conformations of the ATPase domains

Transition between open and closed clamp states is a central feature of the type II topoisomerase reaction cycle (Figure 1, Supplementary Movie S2). Intriguingly, comparison of our open clamp topoisomerase IV structure with the closed structure of yeast topo II determined at 4.4 Å (23) reveals a markedly different disposition of ATPase domains that has potentially important implications (see Figure 9). In the yeast topo II structure, the ATPase domains are locked with ADPNP into a dimerized state in the absence of a T-segment, and the DNA gate is trapped in the cleaved state using a suicide DNA substrate (Figure 9B, PDB entry 4GFH) (23). The structure is suggested to be a snapshot of topo II after the T-segment transport and before the ADP release. Interestingly, in contrast to the open enzyme clamp (Figures 3 and 9A), the ATPase domains of the closed clamp structure are in a criss-cross orientation (bordering a collapsed cavity), which would require rotations of the ATPase domains of >225° in closing the G-gate and then in resetting to the open clamp form (see Figures 8 and 9). If the closed clamp structure represents a reaction intermediate, then transition to a crisscrossed arrangement could act as an irreversible switch ensuring directional DNA transport (23). Such a large conformational change differs from the modest 45^°^ reverse rotation shown in Figure 8, which is attractive in that it allows the enzyme to achieve enzyme turnover with minimal movement and at the same time being supported by the results of biochemical studies of the role of the K-loop master sensor (23).

After our manuscript submission, a cryo-electron microscopy structure was described for an ADPNP-gyrase-DNA complex bound to ciprofloxacin and stabilized by glutaraldehyde cross-linking (50). Based on this closed clamp structure determined at 23 Å resolution, it was suggested that ATPase-TOPRIM domain cross-over occurs to trap the translocated DNA before DNA strand passage (Figure 1, transition **1** to **2**). This arrangement differs from our open clamp topo IV structure at 3.7 Å, which clearly lacks crossovers and is of course a different conformational state in the reaction cycle. Obviously, the poor resolution of cryoEM structures makes it more difficult to trace domain linkage with certainty. However, crossovers could be a feature of type II topoisomerase action that occur at different stages in the reaction trajectory for topo IV, gyrase and topo II. Clearly, high-resolution X-ray structures will be needed to define exactly what conformational transitions occur after clamp closure.

### The open clamp structure and rational design of cleavage-enhancing drugs

Several structures of type II topoisomerase cleavage complexes have been reported recently by us (18,19) and others (16,51,52) comprising the ParE30/ParC55 domains of the topo IV [or equivalent for gyrase (16) and human topo IIβ (52)] with the G-segment DNA bound and stabilized by antibacterials (16,18,19,16,51) or anticancer drugs (52). These structures are the key ‘stepping stones’ for future rational drug design, which will be needed to produce potent antibacterial (and anticancer) therapies that overcome drug resistance. The partial structures reveal very nicely the mode of binding of antibacterial diones (Figure 10A) or quinolones or of anticancer drugs at the DNA gate. However, the absence of ATPase domains leads to some uncertainties in modelling the scope and accessibility of drug binding pockets, as suggested by the closed clamp structure (Figure 9) (23) in which the ATPase domains adopt the criss-cross conformation and partially shield the DNA binding groove from the solvent above. Reassuringly, the open clamp structure of full-length topo IV reveals that the ATPase domains are folded back, and the DNA gate is fully accessible to drugs from solvent above (Figure 10B and in the lower resolution levofloxacin complex, Supplementary Section). As long as the areas immediately close to the ends of the long α-helices of the ATPase domains are avoided, the entire DNA-binding groove within the bound G-segment can be exploited to design new generations of cleavage enhancing drugs (Figure 10). Thus, modelling efforts based on structures of the more readily available cleavage complexes lacking ATPase domains appear to be a reasonable surrogate for the holoenzyme.

## SUMMARY

We have solved the first high-resolution open-clamp structure of a type II topoisomerase-DNA complex complete with N-, DNA- and C-gates containing both bent gate-DNA and a second linear DNA duplex bound at a previously unreported site on ParC. This second DNA duplex reveals the first structure of a gate-DNA before association and bending at the gate. In our open clamp structure, the ATPase domains are folded back and linked to TOPRIM domains on the same side of the enzyme complex, two features that ensure full access for DNA and cleavage enhancing drugs. Based on these structural insights, we propose a mechanism for DNA capture and transport by type II topoisomerases. The work significantly advances understanding of the initial steps by which a macromolecular machine can recognize, capture and transport DNA, a key process in managing the topological consequences of DNA replication, transcription and recombination.

## ACCESSION NUMBERS

4I3H and 4JUO.

## SUPPLEMENTARY DATA

Supplementary Data are available at NAR Online.

Supplementary Data

## References

[1] Gellert M, Mizuuchi K, O'Dea MH, Nash HA (1976). DNA gyrase: an enzyme that introduces superhelical turns into DNA. Proc. Natl Acad. Sci. USA.

[2] Schoeffler AJ, Berger JM (2008). DNA topoisomerases: harnessing and constraining energy to govern chromosome topology. Q. Rev. Biophys..

[3] Vos SM, Tretter EM, Schmidt BH, Berger JM (2011). All tangled up: how cells direct, manage and exploit topoisomerase function. Nat. Rev. Mol. Cell Biol..

[4] Berger JM, Gamblin SJ, Harrison SC, Wang JC (1996). Structure and mechanism of DNA topoisomerase II. Nature.

[5] Corbett KD, Berger JM (2004). Structure, molecular mechanisms, and evolutionary relationships in DNA topoisomerases. Annu. Rev. Biophys. Biomol. Struct..

[6] Corbett KD, Schoeffler AJ, Thomsen ND, Berger JM (2005). The structural basis for substrate specificity in DNA topoisomerase IV. J. Mol. Biol..

[7] Nitiss JL (2009). DNA topoisomerase II and its growing repertoire of biological functions. Nat. Rev. Cancer.

[8] Brown PO, Cozzarelli NR (1979). A sign inversion mechanism for enzymatic supercoiling of DNA. Science.

[9] Mizuuchi K, Fisher LM, O’Dea MH, Fisher LM (1980). DNA gyrase action involves the introduction of transient double-strand breaks into DNA. Proc. Natl Acad. Sci. USA.

[10] Liu LF, Liu CC, Alberts BM (1980). Type II DNA topoisomerases: enzymes that can unknot a topologically knotted DNA molecule via a reversible double-strand break. Cell.

[11] Collin F, Karkare S, Maxwell A (2011). Exploiting bacterial DNA gyrase as a drug target: current state and perspectives. Appl. Microbiol. Biotechnol..

[12] Drlica K, Malik M, Kerns RJ, Zhao X (2008). Quinolone-mediated bacterial death. Antimicrob. Agents Chemother..

[13] Nitiss JL (2009). Targeting DNA topoisomerase II in cancer chemotherapy. Nat. Rev. Cancer.

[14] Pommier Y, Leo E, Zhang H, Marchand C (2010). DNA topoisomerases and their poisoning by anticancer and antibacterial drugs. Chem. Biol..

[15] Fisher LM (1981). DNA supercoiling by DNA gyrase. Nature.

[16] Bax BD, Chan PF, Eggleston DS, Fosberry A, Gentry DR, Gorrec F, Giordano I, Hann MM, Hennessy A, Hibbs M (2010). Type IIA topoisomerase inhibition by a new class of antibacterial agents. Nature.

[17] Dong KC, Berger JM (2007). Structural basis for gate-DNA recognition and bending by type IIA topoisomerases. Nature.

[18] Laponogov I, Sohi MK, Veselkov DA, Pan XS, Sawhney R, Thompson AW, McAuley KE, Fisher LM, Sanderson MR (2009). Structural insight into the quinolone-DNA cleavage complex of type IIA topoisomerases. Nat. Struct. Mol. Biol..

[19] Laponogov I, Pan XS, Veselkov DA, McAuley KE, Fisher LM, Sanderson MR (2010). Structural basis of gate-DNA breakage and resealing by type II topoisomerases. PLoS One.

[20] Kirchhausen T, Wang JC, Harrison SC (1985). DNA gyrase and its complexes with DNA: direct observation by electron microscopy. Cell.

[21] Costenaro L, Grossmann JG, Ebel C, Maxwell A (2005). Small-angle X-ray scattering reveals the solution structure of the full-length DNA gyrase a subunit. Structure.

[22] Baker NM, Weigand S, Maar-Mathias S, Mondragon A (2011). Solution structures of DNA-bound gyrase. Nucleic Acids Res..

[23] Schmidt BH, Osheroff N, Berger JM (2012). Structure of a topoisomerase II-DNA- nucleotide complex reveals a new control mechanism for ATPase activity. Nat. Struct. Mol. Biol..

[24] Pan XS, Fisher LM (1996). Cloning and characterisation of the *parC* and *parE* genes of *Streptococcus pneumoniae*: role in fluoroquinolone resistance. J. Bacteriol..

[25] Pan X-S, Ambler J, Mehtar S, Fisher LM (1996). Involvement of topoisomerase IV and gyrase as ciprofloxacin targets in *Streptococcus pneumoniae*. Antimicrob. Agents Chemother..

[26] Drlica K, Hiasa H, Kerns RJ, Malik M, Mustaev A, Zhao X (2009). Quinolones: action and resistance updated. Curr. Top. Med. Chem..

[27] Pan XS, Fisher LM (1999). *Streptococcus pneumoniae* DNA gyrase and topoisomerase IV: overexpression, purification, and differential inhibition by fluoroquinolones. Antimicrob. Agents Chemother..

[28] Yague G, Morris JE, Pan X-S, Gould KA, Fisher LM (2002). Cleavable complex formation by wild-type and quinolone-resistant *Streptococcus pneumoniae* type II topoisomerases mediated by gemifloxacin and other fluoroquinolones. Antimicrob. Agents Chemother..

[29] Laponogov I, Veselkov DA, Sohi MK, Pan XS, Achari A, Yang C, Ferrara JD, Fisher LM, Sanderson MR (2007). Breakage-reunion domain of *Streptococcus pneumoniae* topoisomerase IV: crystal structure of a gram-positive quinolone target. PLoS One.

[30] Pan X-S, Gould KA, Fisher LM (2009). Probing the differential interactions of quinazolinedione PD 0305970 and quinolones with gyrase and topoisomerase IV. Antimicrob. Agents Chemother..

[31] Leo E, Gould KA, Pan X-S, Capranico G, Sanderson MR, Palumbo M, Fisher LM (2005). Novel symmetric and asymmetric DNA scission determinants for *Streptococcus pneumoniae* topoisomerase IV and gyrase are clustered at the DNA breakage site. J. Biol. Chem..

[32] Arnoldi E, Pan X-S, Fisher LM (2013). Functional determinants of gate-DNA selection and cleavage by bacterial type II topoisomerases. Nucleic Acids Res..

[33] McPherson A, Cudney B (2006). Searching for silver bullets: an alternative strategy for crystallizing macromolecules. J. Struct. Biol..

[34] Kabsch W (2010). Xds. Acta Crystallogr. D Biol. Crystallogr..

[35] Winn MD, Ballard CC, Cowtan KD, Dodson EJ, Emsley P, Evans PR, Keegan RM, Krissinel EB, Leslie AG, McCoy A (2011). Overview of the CCP4 suite and current developments. Acta Crystallogr. D Biol. Crystallogr..

[36] Winter G (2010). xia2: an expert system for macromolecular crystallography data reduction. J. Appl. Cryst..

[37] McCoy AJ, Grosse-Kunstleve RW, Adams PD, Winn MD, Storoni LC, Read RJ (2007). Phaser crystallographic software. J. Appl. Cryst..

[38] Bates PA, Kelley LA, MacCallum RM, Sternberg MJ (2001). Enhancement of protein modeling by human intervention in applying the automatic programs 3D-JIGSAW and 3D-PSSM. Proteins.

[39] Brino L, Urzhumtsev A, Mousli M, Bronner C, Mitschler A, Oudet P, Moras D (2000). Dimerization of *Escherichia coli* DNA-gyrase B provides a structural mechanism for activating the ATPase catalytic center. J. Biol. Chem..

[40] Brunger AT, Adams PD, Clore GM, DeLano WL, Gros P, Grosse-Kunstleve RW, Jiang JS, Kuszewski J, Nilges M, Pannu NS (1998). Crystallography & NMR system: a new software suite for macromolecular structure determination. Acta Crystallogr. D Biol. Crystallogr..

[41] Schroder GF, Levitt M, Brunger AT (2010). Super-resolution biomolecular crystallography with low-resolution data. Nature.

[42] Adams PD, Afonine PV, Bunkoczi G, Chen VB, Davis IW, Echols N, Headd JJ, Hung LW, Kapral GJ, Grosse-Kunstleve RW (2010). PHENIX: a comprehensive Python-based system for macromolecular structure solution. Acta Crystallogr. D Biol. Crystallogr..

[43] Emsley P, Lohkamp B, Scott WG, Cowtan K (2010). Features and development of coot. Acta Crystallogr. D Biol. Crystallogr..

[44] Laskowski RA, MacArthur MW, Moss DS, Thornton JM (1993). PROCHECK - a program to check the stereochemical quality of protein structures. J. Appl. Crystallogr..

[45] Murshudov GN, Skubak P, Lebedev AA, Pannu NS, Steiner RA, Nicholls RA, Winn MD, Long F, Vagin AA (2011). REFMAC5 for the refinement of macromolecular crystal structures. Acta Crystallogr. D Biol. Crystallogr..

[46] Murshudov GN, Vagin AA, Dodson EJ (1997). Refinement of macromolecular structures by the maximum-likelihood method. Acta Crystallogr. D Biol. Crystallogr..

[47] Humphrey W, Dalke A, Schulten K (1996). VMD: visual molecular dynamics. J. Mol. Graph..

[48] Zheng G, Lu XJ, Olson WK (2009). Web 3DNA–a web server for the analysis, reconstruction, and visualization of three-dimensional nucleic-acid structures. Nucleic Acids Res..

[49] Bellon S, Parsons JD, Wei Y, Hayakawa K, Swenson LL, Charifson PS, Lippke JA, Aldape R, Gross CH (2004). Crystal structures of *Escherichia coli* topoisomerase IV ParE subunit (24 and 43 kilodaltons): a single residue dictates differences in novobiocin potency against topoisomerase IV and DNA gyrase. Antimicrob. Agents Chemother..

[50] Papillon J, Menetret J-F, Batisse C, Helye R, Schultz P, Potier N, Lamour V (2013). Structural insight into negative DNA supercoiling by DNA gyrase, a bacterial type 2A DNA topoisomerase. Nucleic Acids Res..

[51] Wohlkonig A, Chan PF, Fosberry AP, Homes P, Huang J, Kranz M, Leydon VR, Miles TJ, Pearson ND, Perera RL (2010). Structural basis of quinolone inhibition of type IIA topoisomerases and target-mediated resistance. Nat. Struct. Mol. Biol..

[52] Wu CC, Li TK, Farh L, Lin LY, Lin TS, Yu YJ, Yen TJ, Chiang CW, Chan NL (2011). Structural basis of type II topoisomerase inhibition by the anticancer drug etoposide. Science.

