# Supplementary material for: Structure of an ‘open’ clamp type II topoisomerase-DNA complex provides a mechanism for DNA capture and transport
Source: Nucleic Acids Res. 2013 Aug 21;41(21):9911–23. doi: 10.1093/nar/gkt749 (PMC3834822; doi:10.1093/nar/gkt749)
Supplement: Supplementary Data [file supp_41_21_9911__index.html]

Structure of an ‘open’ clamp type II topoisomerase-DNA complex provides a mechanism for DNA capture and transport — Structure of an ‘open’ clamp type II topoisomerase-DNA complex provides a mechanism for DNA capture and transport — Supplementary Data 

# Structure of an ‘open’ clamp type II topoisomerase-DNA complex provides a mechanism for DNA capture and transport

## Supplementary Data

files

**Files in this Data Supplement:**

- Supplementary Data - pdf file
- Supplementary Data - mp4 file
- Supplementary Data - mp4 file
