# Supplementary material for: Structure of an ‘open’ clamp type II topoisomerase-DNA complex provides a mechanism for DNA capture and transport
Source: Nucleic Acids Res. 2013 Aug 21;41(21):9911–23. doi: 10.1093/nar/gkt749 (PMC3834822; doi:10.1093/nar/gkt749)
Supplement: Supplementary Data [file supp_gkt749_nar-01692-h-2013-File012.pdf]

Supplementary materials for

Structure of an ‘open’ clamp type II topoisomerase-DNA complex provides a mechanism for DNA capture and transport

Ivan Laponogov[1,2], Dennis A. Veselkov[1], Isabelle M-T. Crevel[2], Xiao-Su Pan[2], L. Mark Fisher[2*] and Mark R. Sanderson[1*]

[1] Randall Division of Cell and Molecular Biophysics, 3rd Floor New Hunt’s House, Division of Medical and Life Sciences, King’s College, Guys Campus, London Bridge, London SE1 1UL, U.K.
[2] Division of Biomedical Sciences, St. George’s, University of London, Cranmer Terrace, London SW17 0RE, U.K.

* To whom correspondence should be addressed. Tel: +44 (0) 207 848 6403; Fax: +44 (0) 207 848 6435; Email: mark.sanderson@kcl.ac.uk. *Correspondence may also be addressed to L. Mark Fisher. Tel: +44 (0) 208 725 5782; Fax: +44 (0) 208 725 2992; Email: lfisher@sgul.ac.uk.

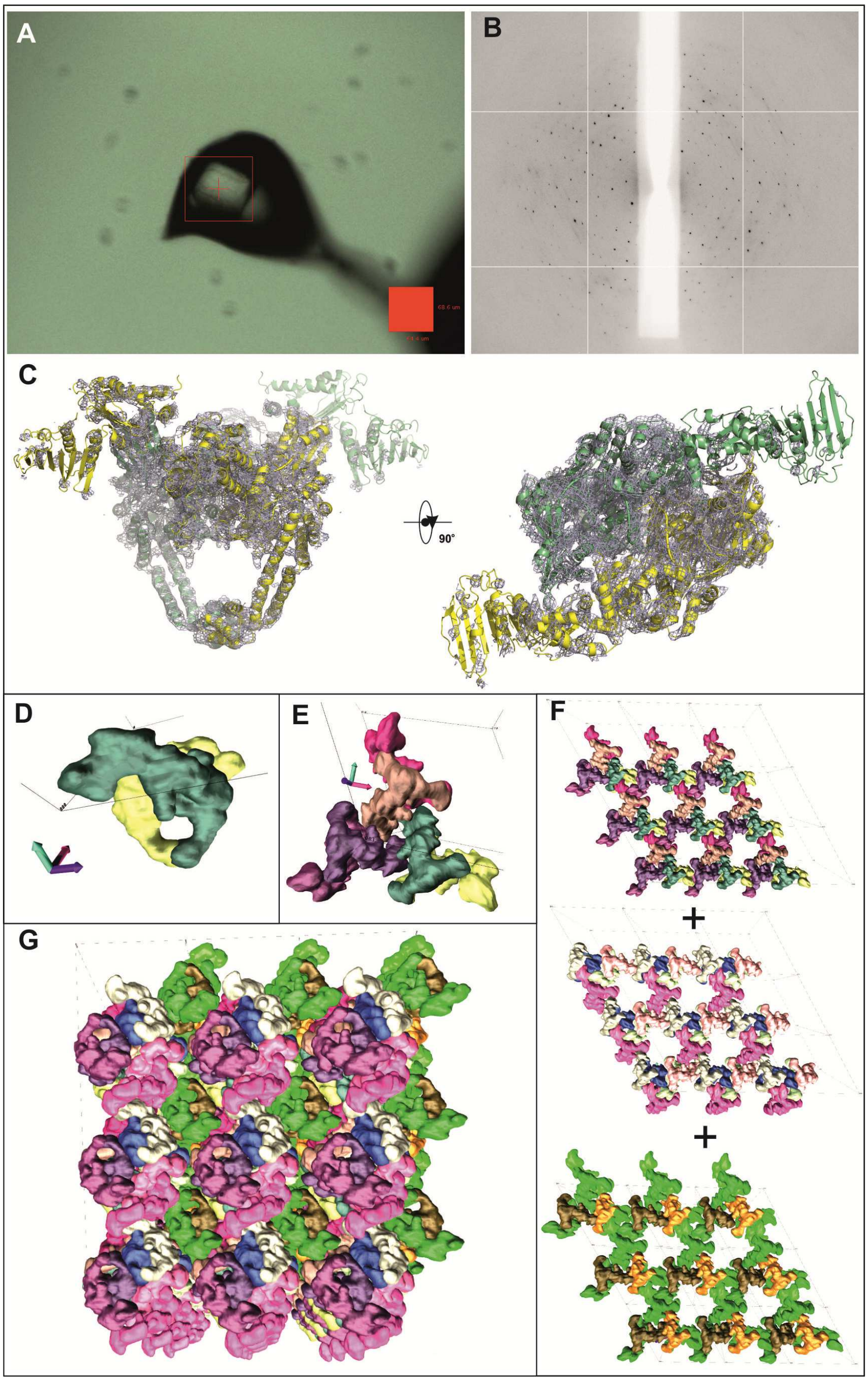

Supplementary Figure 1. Full-length ParE and ParC55 proteins complexed with a 26-mer E-site DNA in space group H32. A, image of the crystal mounted on the goniometer in a cryo-loop on beamline I03 at the Diamond synchrotron (UK) which gave the best resolution of 6.53 Å. **B**, the corresponding diffraction pattern observed for this crystal. **C**, the model of the protein-DNA complex overlapped with the corresponding $2F_{obs}$-F_{calc} map after refinement at 1.2σ level. **D-G**, formation of the crystal lattice in H32 space group: **D**, the asymmetric unit contains only 1/2 of the biological dimer and the second half of the dimer is formed via crystallographic symmetry operations (y+1/3,x+2/3,-z+2/3)+(0,-1,0); **E**, three biological dimers form a "triangle" via intermolecular ParC55-ParE and ParE-ParE interactions; **F**, "triangles" form the hexagonal lattices which, in turn with the corresponding translation operators of (0, 0, 0), (-1/3, +1/3, +1/3) and (1/3, 2/3, 2/3) form the continuous crystal lattice, shown in **G**.

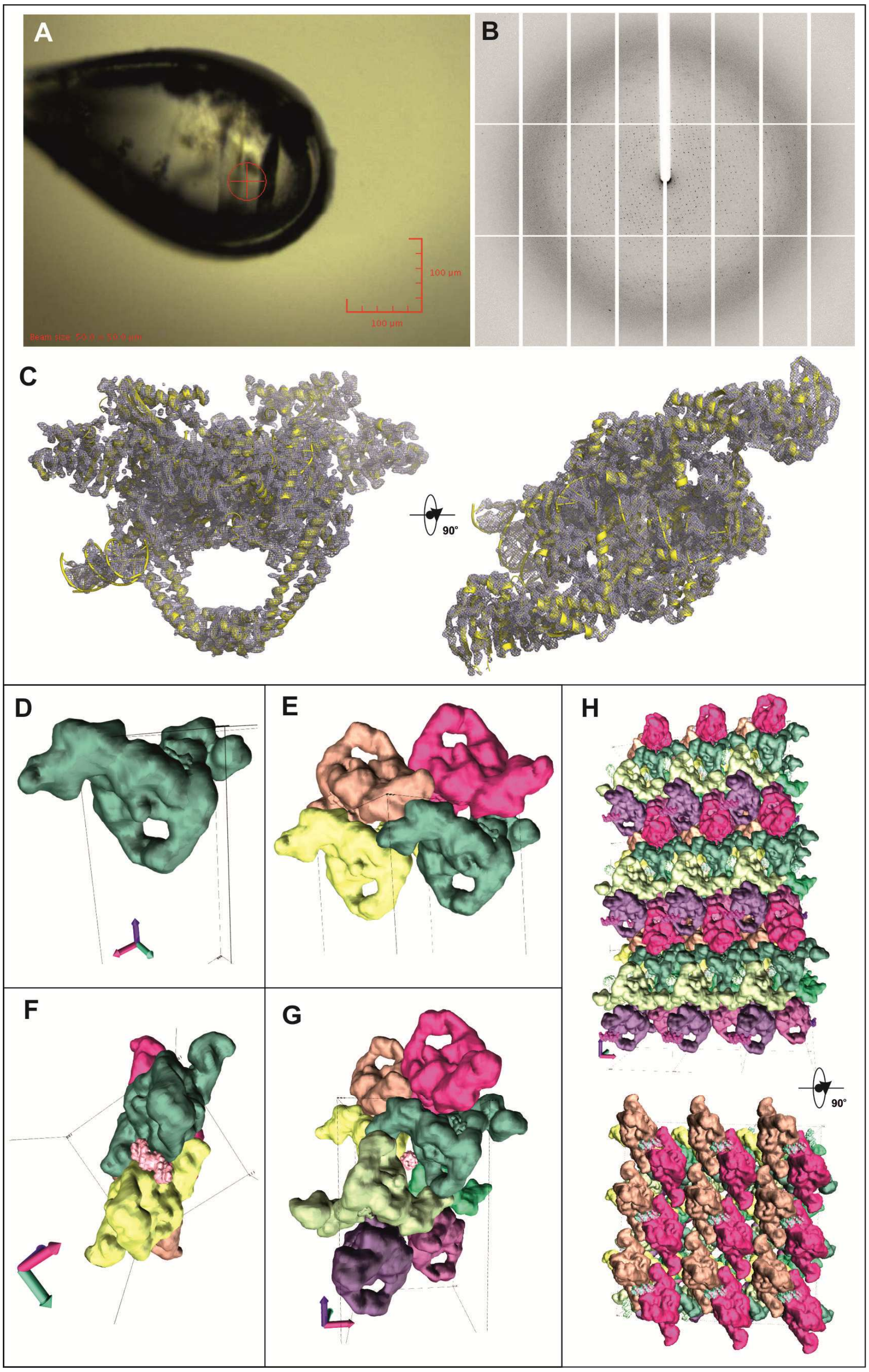

Supplementary Figure 2. Full-length ParE-ParC55 fusion protein complexed with the 34-mer V-site DNA in space group P4$_2$2$_1$2. A, the image of the crystal in a cryo-loop which gave the best resolution of 3.7 Å. **B**, the corresponding diffraction pattern observed for this crystal. **C**, the model of the protein-DNA complex overlapped with the corresponding 2F$_{obs}$-F$_{calc}$ map after refinement at 1.5σ level. **D-H**, formation of the crystal lattice in P4$_2$2$_1$2 space group: **D**, the asymmetric unit contains one biological dimer; **E**, the four dimers are positioned DNA-binding groove to DNA-binding groove in the cell forming large areas of contact between ATPase domains; **F**, the second DNA is bound between the long α-helices of the ParC55 domains close to the C-gate region; **G**, another four biological dimers bind perpendicular to the first ones coordinated via the second DNA (thus forming the 4-fold symmetry of the P4$_2$2$_1$2 space group); **H**, the continuous crystal lattice is formed by the repetition of these operators.

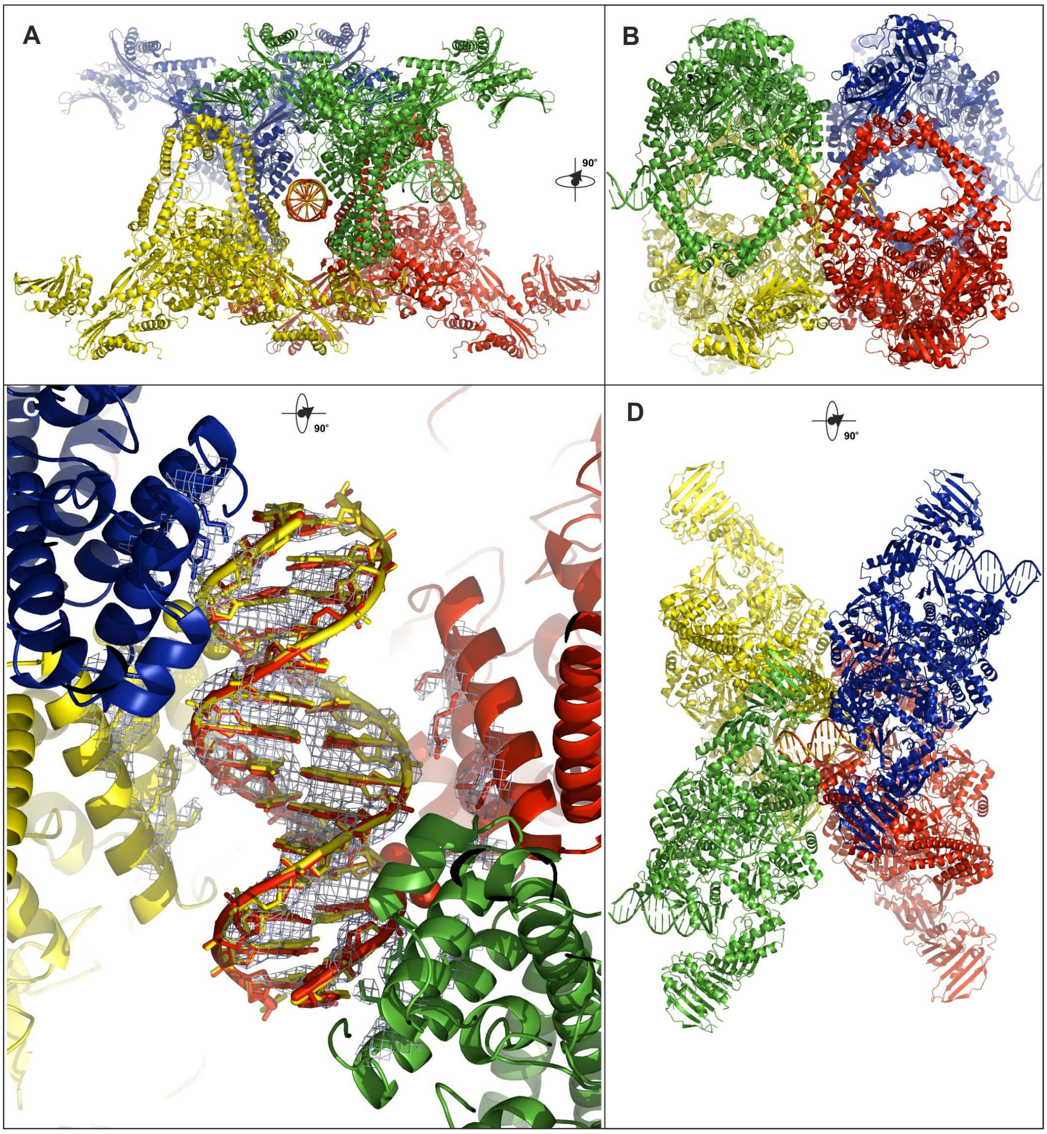

Supplementary Figure 3. Coordination of the four crystallographically related protein-DNA complexes by the second DNA bound to the long α-helices. A, **B** and **D** represent three orthogonal views of the four dimers coordinated by the second DNA. **C** is a "zoom-in" view of the coordination site. Different dimers are shown in different colours (blue, yellow, red and green). The two crystallographically equivalent orientations of the second DNA are shown in red and yellow.

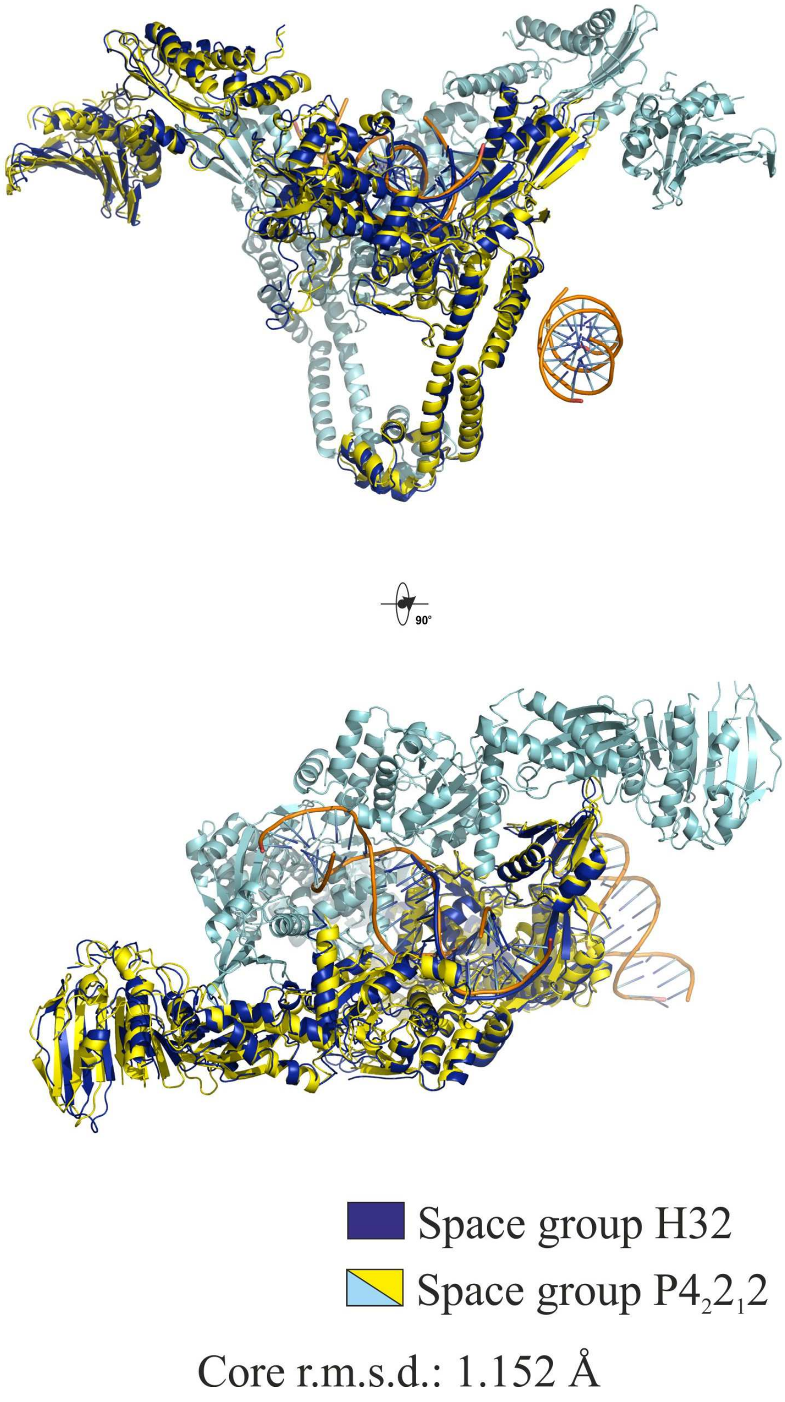

Supplementary Figure 4. Superposition of the full-length ParE/ParC55/DNA structures solved in space groups H32 and $P4_22_12$. The structure in space group $P4_22_12$ is in cyan/yellow whereas the structure in H32 space group is in blue. The core r.m.s.d. was calculated using WinCoot SSM(43).

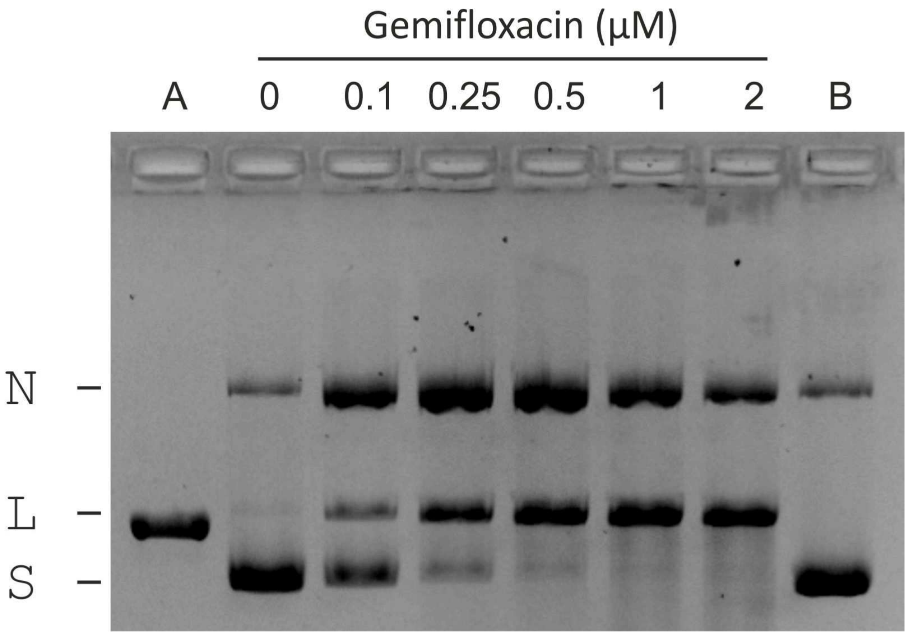

Supplementary Figure 5. Full-length ParE-ParC55 fusion protein is active in DNA cleavage. Supercoiled pBR322 DNA (0.4 μg) was incubated with the *S. pneumoniae* topo IV fusion protein (1 μg) in 40 mM Tris-HCl pH 7.5, 6 mM $MgCl_2$, 10 mM DTT, 200 mM potassium glutamate and 50 μg/ml bovine plasma albumin in the absence or presence of gemifloxacin (final reaction volume 20 μl). After 1 hr at 37°C, the reaction was stopped by addition of SDS to 1% followed by proteinase K to 220 μg/ml and further incubation at 42 ^{o}C for 1h. DNA products were analysed by electrophoresis in a 1% agarose gel. Gemifloxacin concentrations used are shown above the lanes. A, linear pBR322 DNA; B, supercoiled pBR322 incubated with 2 μM gemifloxacin but enzyme omitted. N. L and S denote nicked, linear and supercoiled pBR322 DNA. The fusion protein mediates gemifloxacin-dependent conversion of supercoiled DNA to nicked and linear products.

Supplementary Table 1 Data collection and refinement statistics (**Molecular replacement**)

	Low-resolution ParC55/ParE/E-site 26-mer DNA/ levofloxacin complex	High-resolution ParC55-ParE/V-site 34-mer DNA complex
**Data collection**		
Space group	H32	$P4_22_12$
Cell dimensions		
a, *b*, *c* (Å)	213.58, 213.58, 211.83	160.60, 160.60, 280.56
α, β, γ (°)	90, 90, 120	90, 90, 90
Resolution (Å)	53.4-6.53 (6.71-6.53)*	72.19-3.70 (3.90-3.70)*
R_{sym} or R_{merge}	0.06 (0.41)	0.12 (0.45)
$I/\sigma I$	6.84 (1.88)	5.23 (1.71)
Completeness (%)	99.24 (99.66)	99.75 (100)
Redundancy	9.66 (l0.93)	12.31 (11.46)
**Refinement**		
Resolution (Å)	46.44-6.53	71.82-3.70
No. reflections	3701	39825
$R_{work/}$ R_{free}	24.74%/29.82%	18.55%/24.85%
No. atoms		
Protein	7506	14849
Ligand/ion	47	3
Water	0	2
B-factors		
Protein	192.71	87.08
Ligand/ion	97.75	92.09
Water	-	63.24
R.m.s deviations		
Bond lengths (Å)	0.017	0.009
Bond angles (º)	1.491	1.396

*Highest resolution shell is shown in parenthesis.

Movie S1. Overall views of the high-resolution structure of the open ATP-gate complex of topoisomerase IV from *S. pneumoniae*. The protein is shown in cartoon representation, the DNA G-segment is in surface representation. The colour scheme is as in the corresponding Figure 3 within the main text of the paper.

Movie S2. The proposed model for the T-segment DNA capture and transition based on the high-resolution structure of the open N-gate topoisomerase IV complex with the V34 G-segment DNA. The movie shows the transition of the T-segment according to our crystallographic results and modelling based on the principle of the minimal movement and distortion of the domains for the sake of performing the task. The colour scheme is as in the corresponding Figure 3 within the main text of the paper.
